# Recent advances in quantum nanophotonics: plexcitonic and vibro-polaritonic strong coupling and its biomedical and chemical applications

**DOI:** 10.1515/nanoph-2022-0542

**Published:** 2022-11-11

**Authors:** Yangkyu Kim, Aleksandr Barulin, Sangwon Kim, Luke P. Lee, Inki Kim

**Affiliations:** Department of Biophysics, Institute of Quantum Biophysics, Sungkyunkwan University, Suwon 16419, Republic of Korea; and Department of Intelligent Precision Healthcare Convergence, Sungkyunkwan University, Suwon 16419, Republic of Korea; Department of Medicine, Harvard Medical School, Brigham and Women’s Hospital, Boston, MA 02115, USA; Department of Bioengineering, Department of Electrical Engineering and Computer Science, University of California, Berkeley, Berkeley, CA 94720, USA

**Keywords:** chemical reaction, plexcitons, quantum biological process, strong coupling, vibro-polaritonic strong coupling

## Abstract

The fundamental understanding of molecular quantum electrodynamics via the strong light–matter interactions between a nanophotonic cavity and quantum emitters opens various applications in quantum biology, biophysics, and chemistry. However, considerable obstacles to obtaining a clear understanding of coupling mechanisms via reliable experimental quantifications remain to be resolved before this field can truly blossom toward practical applications in quantitative life science and photochemistry. Here, we provide recent advancements of state-of-the-art demonstrations in plexcitonic and vibro-polaritonic strong couplings and their applications. We highlight recent studies on various strong coupling systems for altering chemical reaction landscapes. Then, we discuss reports dedicated to the utilization of strong coupling methods for biomolecular sensing, protein functioning studies, and the generation of hybrid light–matter states inside living cells. The strong coupling regime provides a tool for investigating and altering coherent quantum processes in natural biological processes. We also provide an overview of new findings and future avenues of quantum biology and biochemistry.

## 1 Introduction

When photons strike a material, the interactions result in first- or second-order optical linear and nonlinear processes such as reflection, absorption, transmission, scattering, and emission. Perturbations caused by electromagnetic fields in the material usually are not considered as long as the interaction remains weak. Nevertheless, the enhanced light–matter interactions inside optical resonators may enter the strong coupling regime, which perturbs or fundamentally modifies the chemical and physical properties of the material, which become tied together with the light–matter coupling strength. Advances in cavity quantum electrodynamics (cavity QED) can offer insight into the relationship between atomic states of matter and electromagnetic modes from a quantum perspective [1].

The strong coupling has been employed to moderate the rate and thermodynamics of chemical reactions for enhanced reaction selectivity [2] and catalysis [3, 4]. The field of strong coupling–assisted chemistry is gradually emerging as a new tool to transform chemical landscapes. In biology, photosynthesis benefits from quantum coherence and dephasing and involves these effects for enhanced energy transport to the reaction centers of light-harvesting complexes [5]. Strong coupling provides a way to probe these quantum coherence effects and produce new insight into the energy transfer mechanisms. Creating hybrid light–matter states inside a living cell is an exciting prospect for biology, as it may affect or even promote cell growth inside the optical resonator [6]. In contrast, plasmonic nanostructures ensure the ability to confine an electromagnetic field in nanometer-size cavities and achieve plexcitonic strong coupling with even individual quantum emitters (QEs) [7], which is pertinent for quantum biosensing applications or single atom–single photon interaction studies.

In this review, we discuss the main light–matter coupling regimes in optical resonators and experimental implementations of plexcitonic and vibro-polaritonic strong coupling (or vibrational strong coupling (VSC)). Next, we provide an overview of the systems adopted for modification of chemical reaction selectivity and catalysis under vibro-polaritonic strong coupling. Then, we emphasize reports on the role of strong coupling in modifying enzyme catalytic activity, probing the coherent energy transfer within light-harvesting complexes, and generating hybrid light–matter states inside living cells. We conclude with an outlook of biological processes that involve complex electron transfer pathways and could be interrogated under the strong coupling regime.

## 2 Fundamentals of strong coupling

### 2.1 Light–matter coupling regimes

The field of cavity QED has revolutionized the ability to manipulate light–matter interactions. Cavity QED aims to control the coupling strength between a QE and photon modes as well as irreversible losses of photons inside the cavity [8]. The possibility of tailoring the electromagnetic environment surrounding the emitter prompted the development of various resonators with given *Q*-factors and mode volumes for coupling with simple two-level systems [9, 10]. The interaction strength and losses of the hybrid systems dictates whether the system is in a weak or strong light–matter coupling regime.

#### 2.1.1 Weak coupling

The weak coupling regime is principally associated with the accelerated decay rate of the emitter two-level system due to confined excitation light called the Purcell effect [11]. The Purcell effect strongly depends on the quality factor and mode volume of the cavity [11, 12] (Equation (1)),

(1) $$F_{p}\left(\omega \right)=\frac{6\pi c^{3}Q}{\omega^{3}V_{m}}∼\frac{\Gamma }{\Gamma_{0}}.$$

Here, *c* is the speed of light*,* *Q* is the quality factor of the cavity, *ω* is the electric dipole frequency, and *V*_*m*_ is the mode volume of the cavity, while Γ and Γ_0_ denote the total decay rates of the emitter in the presence and absence of an optical cavity, respectively.

The local density of electromagnetic states (LDOS) of the emitter, which quantifies the energy dissipation of an electric dipole in a nanoenvironment, becomes modified in the presence of a nanophotonic structure [13]. Due to the electromagnetic field manipulation in the weak coupling regime, many advances have been made in ensemble and single-molecule sensing [14–18], fast nanoscale emitters [19], on-demand single-photon devices [20], and low-threshold solid state lasers [21]. Nevertheless, in the weak coupling regime, the loss of photons escaping the cavity dominates the coupling strength with the emitter [22]. The light and cavity modes remain decoupled, and the fundamental quantum mechanical single atom–single photon processes cannot be probed or manipulated.

#### 2.1.2 Strong coupling

**Figure 1:**
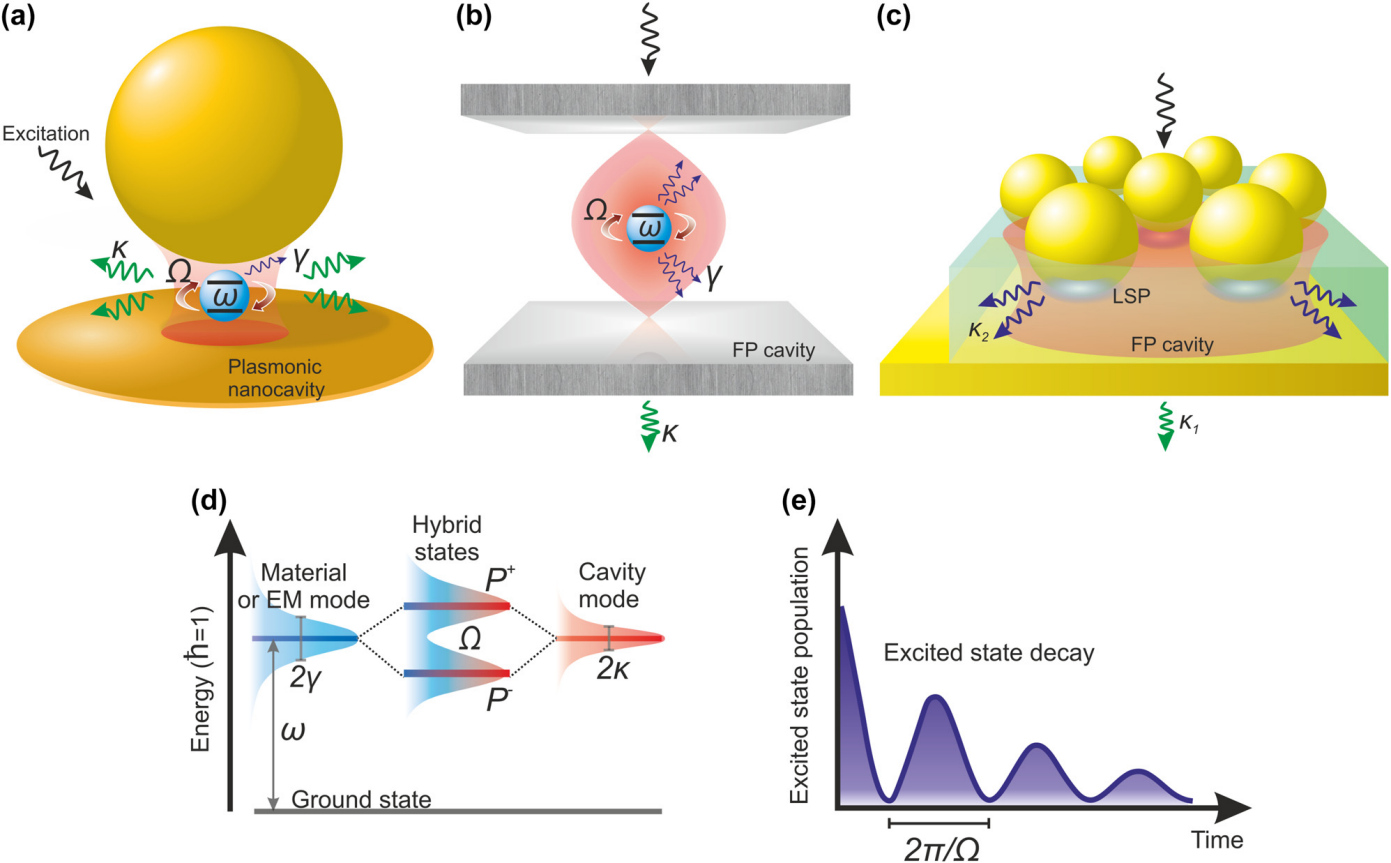
Strong coupling scheme. Examples of (a) plexcitonic, (b) polaritonic, and (c) modal strong coupling systems. Ω corresponds to Rabi splitting, *ω* is the transition frequency of the emitter, and *γ* and *κ* denote emitter and cavity losses, respectively. In panel (c) *κ*_1_ and *κ*_2_ are losses related to two resonant modes of the modal strong coupling. (d) Energy diagram of the coupled system with the corresponding Rabi splitting. P^+^ and P^−^ denote the upper and lower hybrid states, respectively. (e) Rabi oscillations of the excited state population of the two-level system in the time domain.

The strong coupling regime makes it possible to study coherent quantum dynamics between light and matter [23]. Strong coupling occurs when the coupling strength (*g*) between an emitter and a cavity prevails over the photon leakage rate (*κ* = *ω*_*c*_/*Q*, *ω*_*c*_ is the frequency of the cavity mode) and the nonradiative losses of the emitter (*γ*) as follows: 2*g* > *κ* + *γ* [22, 24]. Currently, optical systems that enable observing the strong coupling regime include plasmonic nanostructures, dielectric and Fabry–Pérot microcavities, and modal strong coupling systems [9, 25], [26], [27] (Figure 1a–c). The hybridization between a two-level system and a cavity mode leads to the formation of light–matter energy “dressed” states (Figure 1d). These states are associated with Rabi splitting (*Ω*), which amounts to $\sqrt{4g^{2}-{\left(\gamma -\kappa \right)}^{2}}$ , provided that the cavity and emitter resonance frequencies match [22, 28]. The photon emitted inside the cavity has a significant probability of being absorbed by the emitter before it leaves the cavity, leading to reversible coherent energy transfers of rate *Ω* between light and matter, also called Rabi oscillations (Figure 1e) [28, 29].

Conventionally, the quantum Rabi model describes the interactions between a single two-level atom and a monochromatic electromagnetic mode [30]. The Hamiltonian of the coupled system ( ${\hat{H}}_{\text{QRM}}$ ) can be expressed as

(2) $$\begin{array}{ll} {\hat{H}}_{\text{QRM}} & =\frac{\hbar \omega }{2}{\hat{\sigma }}_{z}+\hbar \omega_{c}{\hat{a}}^{†}\hat{a}+\hbar g_{1}\left(\hat{a}{\hat{\sigma }}_{+}+{\hat{a}}^{†}{\hat{\sigma }}_{-}\right)\\ & \;+\hbar g_{2}\left(\hat{a}{\hat{\sigma }}_{-}+{\hat{a}}^{†}{\hat{\sigma }}_{+}\right), \end{array}$$

where *ω* and *ω*_*c*_ are respective frequencies of the two-level system transition and cavity mode, ${\hat{a}}^{†}$ and $\hat{a}$ denote creation and annihilation operators of the cavity mode, *g*_1_ and *g*_2_ are the light–matter coupling strengths of the resonant and antiresonant interaction terms, and ${\hat{\sigma }}_{z}$ and $\hat{\sigma }$ _*x*_ = $\hat{\sigma }$ _−_ + $\hat{\sigma }$ _+_ are Pauli matrices that describe the atomic spin [23]. In the quantum Rabi model, the coupling strength of the resonant term is equal to that of the antiresonant term (*g* = *g*_1_ = *g*_2_). However, this condition is not maintained under the anisotropic Rabi model [31]. In the rotating-wave approximation (RWA), the antiresonant term $\hbar g_{2}\left(\hat{a}{\hat{\sigma }}_{-}+{\hat{a}}^{†}{\hat{\sigma }}_{+}\right)$ is omitted for *g*/*ω* << 1 and *ω* ≈ *ω*_*c*_ [32], yielding the Jaynes–Cummings model [33].

To reach the strong coupling regime, the dipole moment of the QE, its orientation, and the mode volume of the cavity must be carefully controlled. The optical resonator frequency *g* between a cavity mode and a two-level atom can be written as follows [34, 35]:

(3) $$g=\sqrt{\frac{\left|\vec{\varepsilon }\cdot {\vec{\mu }}_{0}\right|\omega_{c}}{2\hbar \varepsilon_{0}V_{m}}}$$

Here, $\vec{\varepsilon }$ is the polarization vector, ${\vec{\mu }}_{0}$ is the dipole moment of the atom transition, and *ɛ*_0_ is the vacuum permittivity. The transition dipole moment that sets the oscillator strength varies from a few to a hundred Debye with conventional emitters such as quantum dots, organic molecules, and NV-centers in diamond [36]. The mode volume of dielectric and Fabry–Pérot cavities cannot be reduced below (*λ*/2)^3^ ∼ 10^−18^ m^3^ due to the diffraction limit [37], which sets an intrinsic limitation on the coupling strength. Plasmonic nanocavities provide an advantage, as the mode volume can be reduced to a few cubic nanometers [7, 38], although plasmonic nanocavities yield substantial losses and modest quality factors [39]. Nevertheless, the dramatic reduction of nanocavity mode volumes allows the experimental realization of the strong coupling regime with dozens of emitters [40] and even single quantum dots [37, 41], [42], [43] or single-molecule excitons [7]. Moreover, cryogenic temperatures facilitate the achievement of a strong coupling regime in cavity QED experiments due to the reduction of emitter linewidth [44–46], which increases the surface plasmon–polariton propagation length of plasmonic cavities [8]. Experimentally, the Rabi splitting in the cavity QED can be validated based on transmittance [47–49], scattering [37, 41], absorbance [44, 50], reflectance [51, 52], or photoluminescence (PL) [42, 43, 53] spectra.

The Rabi splitting can be boosted proportionally to the square root of the number of emitters that contribute to the coupling ( $h\Omega =h\Omega_{i}\sqrt{N}$ where Ω_*i*_ is the contribution of each emitter in the overall energy splitting) [28]. The extension of the quantum Rabi model to the interaction between the collective dipole and the cavity is represented by either the Dicke model or the Hopfield model, which transition to the Tavis–Cummings model in the RWA [30]. The Hamiltonian of the generalized Hopfield model can be written as [30, 50]

(4) $$\begin{array}{ll} {\hat{H}}_{\text{Hopfield}} & =\hbar \omega_{c}{\hat{a}}^{†}\hat{a}+\hbar \omega {\hat{b}}^{†}\hat{b}+i\hbar g_{1}\left(\hat{a}{\hat{b}}^{†}-{\hat{a}}^{†}\hat{b}\right)\\ & \;+i\hbar g_{2}\left({\hat{a}}^{†}{\hat{b}}^{†}-\hat{a}\hat{b}\right)+{\hat{H}}_{A^{2}} \end{array}$$

where $\omega ,{\hat{b}}^{†}$ , and $\hat{b}$ are frequency, creation, and annihilation operators of the collective excitations of matter, respectively, while ${\hat{H}}_{A^{2}}=\hbar D{\left(\hat{a}+{\hat{a}}^{†}\right)}^{2}$ is the diamagnetic or *A*^2^ term (*D* ≥ *g*^2^/*ω*). The contribution of the counter-rotating and diamagnetic terms is considerable only if *g* amounts to a substantial fraction of the value of *ω*.

#### 2.1.3 Intermediate coupling

When the coupling strength is between strong and weak coupling, the regime is called intermediate coupling (2*g* ∼ *γ* + *κ*) [39]. Normally, this coupling regime is characterized by energy level splitting, which is comparable to the cavity mode or the emitter linewidth. In the intermediate coupling regime, the Fano interferences become experimentally observable based on coherent optical processes, *e.g.*, scattering [37]. Therefore, the measurement of PL spectra as an incoherent process may be more robust to differentiate strong and intermediate coupling regimes experimentally.

The intermediate coupling regime features a strong excitonic effect that could be interesting for 2D dichalcogenide material investigations, as they possess remarkable optical and optoelectronic properties, a large dipole moment, and a photostability [54, 55]. To probe the coupling with 2D materials, one may take advantage of a nanoparticle-on-mirror (NPoM) cavity that provides sub-nanometer tunability of the nanocavity length [19, 54]. The photoluminescence of WSe_2_ monolayers in a nanocavity can be enhanced up to 1700 times compared to a free space [39]. The enhanced PL takes advantage of both the plasmonic field confinement and the strong excitonic effect, which are not accessible simultaneously in the weak or strong coupling regime.

#### 2.1.4 Ultrastrong coupling

Ultrastrong coupling (USC) is a regime where the coupling strength reaches a significant fraction that exceeds 10% of the transition energy and is characterized by the normalized coupling strength value of *g*/*ω* [22]. The rotating-wave approximation breaks down in the USC regime, and the counter-rotating terms that account for the simultaneous creation of correlated light and matter excitation as well as the *A*^2^-term cannot be neglected (Equations (2) and (4)) [23]. The generalized quantum Rabi or Hopfield models provide the correct evolution of energy levels in the USC regime [30]. The corresponding shift of resonant frequency of the original Hamiltonian as compared to the prediction with RWA is attributed to the Bloch–Siegert shift [56]. The ground state behaves as squeezed vacuum and can be populated with virtual photons [30]. These virtual photons are bound in the matter, though there have been several attempts to extract these photons via processes reminiscent of the dynamic Casimir effect [57, 58].

The USC regime has been experimentally observed in superconducting circuits [59–61], intersubband and Landau polaritons [62, 63], organic molecules [52, 64, 65], phonons [66, 67], and transverse plasmons in 3D plasmonic nanoparticle crystals [50]. USC may be obtained outside the strong coupling regime if the linewidths of the cavity and matter represent a significant fraction of the transitions themselves [57]. Experimental demonstration of USC of single QEs in a cavity QED seems highly challenging due to low dipole moments and high required values of *g*/*ω*. Theoretical feasibility has been shown for plasmonic systems with mode volumes below 10 nm^3^ [68]. Superconducting circuits are the only single two-level systems that reach the ultrastrong light–matter coupling regime, as the circuit-QED systems do not require collective excitations [23, 59].

Applications of the USC regime may enable the observation of new quantum effects and applications. The conductivity of n- and p-type semiconductors can be enhanced under USC due to the valence band modification in the USC regime [64, 69]. The USC regime may influence scattering properties leading to a nonlinear frequency conversion [70]. Photodynamic acceleration beyond the strong coupling regime may enable even faster devices for quantum computing to observe quantum gates [71]. The observation of entangled hybrid light–matter ground states is an example of new stable states of matter, which can be further facilitated in the deep strong coupling regime [59].

#### 2.1.5 Deep strong coupling

The deep strong coupling (DSC) regime is defined by extreme values of the normalized coupling strength above the transition energy. The light and matter become decoupled, as the increased contribution of the ${\hat{H}}_{A^{2}}$ term (Equation (4)) acts as a potential barrier leading to reduced radiative damping or the Purcell effect breakdown [50, 72]. Considering that the population of virtual photons in the ground state grows drastically in the DSC regime, fast Rabi splitting modulation of a superposition of multiple resonators may release these photons and permit detectable quantum vacuum radiation in the future [63].

DSC has been experimentally achieved with superconducting qubit-LC oscillator circuits [59] and Landau polaritons at low temperatures [63], whose oscillator strengths are tunable within the GHz or THz range. Recently, unprecedented Rabi splitting above 3 eV was achieved by coupling transverse plasmons in three-dimensional gold nanoparticle crystals to free-space photons, which was the first demonstration of DSC of electronic excitation of a material at room temperature [50]. A normalized coupling strength of 1.83 was reported, giving unique possibilities to explore the Purcell effect breakdown, evaluate the Block–Siegert shift, or compare the experimental applicability of known quantum models of collective bosonic excitations. The DSC regime could enable additional quantum phenomena such as quantum vacuum phase transitions, quantum vacuum radiation, or ground-state modifications for chemistry under strong coupling.

### 2.2 Plexcitons

Plasmonic nanostructures can confine and control the light in the nanometer range, forming plasmonic resonance and adjusting the frequency of the resonance by changing the shape and size of the structure. If excitons exist in the vicinity of plasmonic nanocavity, strong coupling between a plasmon and an exciton yields a quasi-particle “plexciton” [73]. Plexcitons are modeled by a single resonant mode of a cavity of plasmonic nanostructures and by an exciton with a two-level system in the quantum regime [74]. The Hamiltonian of this system without damping includes three parts: *H* = *H*_pl_ + *H*_ex_ + *H*_int_. Each term is the Hamiltonian of the plasmon, the exciton, and the interaction between the plasmon and the exciton. This Hamiltonian of this system can be described as the Jaynes–Cummings (JC) model [33, 74, 75] (Equation (2) with *ω* ≡ *ω*_ex_ the exciton transition frequency, *ω*_*c*_ ≡ *ω*_pl_ the plasmon resonance frequency, and *g*_2_ = 0).

The frequency of the hybrid state obtained through diagonalization of the JC model is given as [74, 76],

(5) $$\omega_{\pm }=\frac{\left(\omega_{\text{pl}}+\omega_{\text{ex}}\right)}{2}\pm \frac{1}{2}\Omega_{\text{plex}},$$

where the splitting of upper and lower plexciton branches is $\Omega_{\text{plex}}=\sqrt{4g^{2}+\delta^{2}}$ with *δ* = *ω*_pl_ − *ω*_ex_ as the detuning parameter. Both the plasmon and exciton feature damping rates of *κ*_pl_ and *γ*_ex_, respectively [9]. Various methods are introduced to take into account incoherent processes caused by damping [24]. Phenomenologically, the following non-Hermitian Hamiltonian considers complex frequencies of the exciton and the plasmonic nanocavity [24]:

(6) $$H_{\text{loss}}=\left[\begin{array}{cc} \omega_{\text{ex}}-i\gamma_{\text{ex}} & g\\ g & \omega_{\text{pl}}-i\kappa_{\text{pl}} \end{array}\right],$$

At zero detuning, the frequency of two states of the plexciton through diagonalization is given as follows [24]:

(7) $$\omega_{\pm }=\frac{\omega_{\text{pl}}+\omega_{\text{ex}}-i\left(\gamma_{\text{ex}}+\kappa_{\text{pl}}\right)\pm \sqrt{4g^{2}-{\left(\gamma_{\text{ex}}-\kappa_{\text{pl}}\right)}^{2}}}{2}.$$

where the Rabi frequency is given by:

(8) $$\Omega =\sqrt{4g^{2}-{\left(\gamma_{\text{ex}}-\kappa_{\text{pl}}\right)}^{2}}.$$

Plexcitonic strong coupling presence can be validated when the coupling strength satisfies the following conditions:

(9) $$2g>\left|\gamma_{\text{ex}}-\kappa_{\text{pl}}\right|,$$

(10) $$\Omega >\gamma_{\text{ex}}+\kappa_{\text{pl}}$$

In addition to the cavity, the influence of surface plasmon (SP) should be considered for describing plexcitonic strong coupling. SPs mostly consist of surface plasmon polaritons (SPPs) and localized surface plasmon (LSP). Waks et al. [77] described the interaction between MNP (metallic nanoparticles) with LSP and emitter in quantum optics. Peng et al. [78] developed a method involving a system that includes a cavity, an MNP, and an emitter. The Hamiltonian of the cavity-engineered MNP-emitter system is divided into a noninteraction part and an interaction part, *H* = *H*_0_ + *H*_int_. Assuming an MNP as a plasmon and an emitter as an exciton, the noninteraction parts and interaction part are given by [78]

(11) $$H_{0}=\omega_{c}{\hat{c}}^{†}\hat{c}+\omega_{pl}{\hat{a}}^{†}\hat{a}+\frac{1}{2}\omega_{ex}{\hat{\sigma }}_{z},$$

(12) $$\begin{array}{ll} H_{\text{int}} & =g_{1}\left({\hat{a}}^{†}\hat{c}+\hat{a}{\hat{c}}^{†}\right)+g_{2}\left({\hat{a}}^{†}{\hat{\sigma }}_{-}+\hat{a}{\hat{\sigma }}_{+}\right)\\ & \;+g_{3}\left({\hat{c}}^{†}{\hat{\sigma }}_{-}+\hat{c}{\hat{\sigma }}_{+}\right), \end{array}$$

where $\hat{c}$ is the annihilation operator of the cavity mode with resonance frequency *ω*_*c*_, *g*_1_ is the coupling coefficient between the plasmon and cavity, and *g*_2_(*g*_3_) represents the coupling coefficient between the exciton and the plasmon (cavity). The quantum Langevin equation is used to describe loss mechanisms, such as damping and radiation. The quantum Langevin equation is induced as follows [78],

(13) $$\frac{d\hat{c}}{dt}=-\left(i\omega_{c}+\frac{\kappa_{c}}{2}\right)\hat{c}-ig_{1}\hat{a}-ig_{3}{\hat{\sigma }}_{-}+{\hat{F}}_{c},$$

(14) $$\frac{d\hat{a}}{dt}=-\left(i\omega_{\text{pl}}+\frac{\kappa_{\text{pl}}}{2}\right)\hat{a}-ig_{1}\hat{c}-ig_{2}{\hat{\sigma }}_{-}+{\hat{F}}_{\text{pl}},$$

(15) $$\begin{array}{ll} \frac{d{\hat{\sigma }}_{-}}{dt} & =-\left(i\omega_{ex}+\frac{\gamma_{\text{ex}}}{2}\right){\hat{\sigma }}_{-}+i\left(g_{2}\hat{a}+g_{3}\hat{c}\right){\hat{\sigma }}_{z}-\sqrt{\gamma_{\text{in}}}{\hat{\sigma }}_{\text{in},-}\\ & \;+{\hat{F}}_{\text{ex}}, \end{array}$$

where ${\hat{F}}_{c}$ , ${\hat{F}}_{pl}$ , and ${\hat{F}}_{ex}$ are noise operators, *γ*_in_ is the rate of the optical pump to the exciton, input operator ${\hat{\sigma }}_{in,-}$ presents the optical pump to the exciton, and *κ*_*c*_ is the decay rate of the cavity. In addition, the QNM model (quasi-normal modes) based on the plasmonic system, which considers absorption and radiation dissipation, was developed into the QNM-JC model by Frank et al. [9, 79, 80].

### 2.3 Vibro-polaritons

A phonon represents a quasiparticle of excited quantized vibrational modes of periodic lattices in condensed matter [81]. Phonons are essential in interpreting thermal conductivity, enthalpy, entropy, and neutron scattering in solid systems [82]. Similar to phonons, individual molecule vibrations can be excited with electromagnetic radiation leading to the stretching or bending of particular chemical bonds [83]. Molecular vibrations are crucial in reaction dynamics and energy transfer and underlie the fluorescence and Raman properties [84]. A result of the strong coupling of cavity modes with phonons or vibrational transitions of molecules in the material gives rise to the formation of the “vibro-polaritons” [85]. Vibro-polaritons provide novel tools to alter chemical reaction kinetics [83], modify the energy levels in biomolecules [86], or manipulate phonon polariton spectra in crystals [67].

Strong coupling with vibrational modes is associated with small oscillator strengths and mid-infrared resonance wavelengths [66]. For example, split ring resonators enable strong coupling with phonon bands of thin films of SiO_2_ at 130 meV (∼31 THz) [87]. Plasmonic nanoantennas covered with phononic layers of matching resonances may exhibit higher transparency that strongly depends on the phonon polariton coupling strength [88]. Taking advantage of the low mode volume, terahertz plasmonic nanocavities make it possible to reach strong coupling with Fröhlich phonon resonances of CdSe nanocrystals [89]. Strong coupling of phonon polaritons with molecule vibrational transitions makes sensitive and compact devices for strong coupling thanks to extreme mode compression of phonons. Hyperbolic phonon polariton resonators based on hyperbolic boron nitride (h-BN) ribbons provide excellent quality factors. They can achieve strong coupling regime with vibrational modes of organic semiconductor CBP at wavenumbers between 1400 and 1550 cm^−1^ [26]. The propagating phonon polaritons have also been reported to couple with atomically thin CBP molecules in real-space [90]. Quartz micropillar-based surface phonon polariton resonators feature smaller mode volumes than h-BN and can reach vibro-polaritonic strong coupling with only a few thousand of 4-nitrobenzyl alcohol molecules [91].

Ultrastrong coupling with vibrational modes has recently been reported in experimental studies. Iron pentacarbonyl molecules have been coupled with Fabry–Pérot cavity modes leading to heavy polaritonic states and reduced vibro-polaritonic linewidths compared to the uncoupled cavity and vibrational modes, which indicates enhanced coherence time [92]. Transverse optical phonons of h-BN layers were coupled with gold microcavity modes [67]. Tight field confinement within hotspot volumes of *λ*^3^/10^7^ in plasmonic epsilon-near-zero cavities enables the ultrastrong coupling with phonons of SiO_2_ lattice with *g*/*ω* values above 0.25 [66].

## 3 Configuration and characteristics of plexcitons

### 3.1 Plexcitonic nanostructure configuration

With the development of nanostructure manufacturing technology, strong coupling systems include diverse plasmonic nanostructures, from simple film layer structures to complex hybrid structures combined with various structures [9, 23, 24, 28, 29, 35, 36, 73, 76, 93], [94], [95], [96]. Plasmon nanostructures with adequate mode volumes showing high coupling strength even at the level of single emitters and single metal nanoparticles at room temperature have recently been studied. Alternatively, plexcitonic systems have been developed to observe and understand strong coupling characteristics effectively.

**Figure 2:**
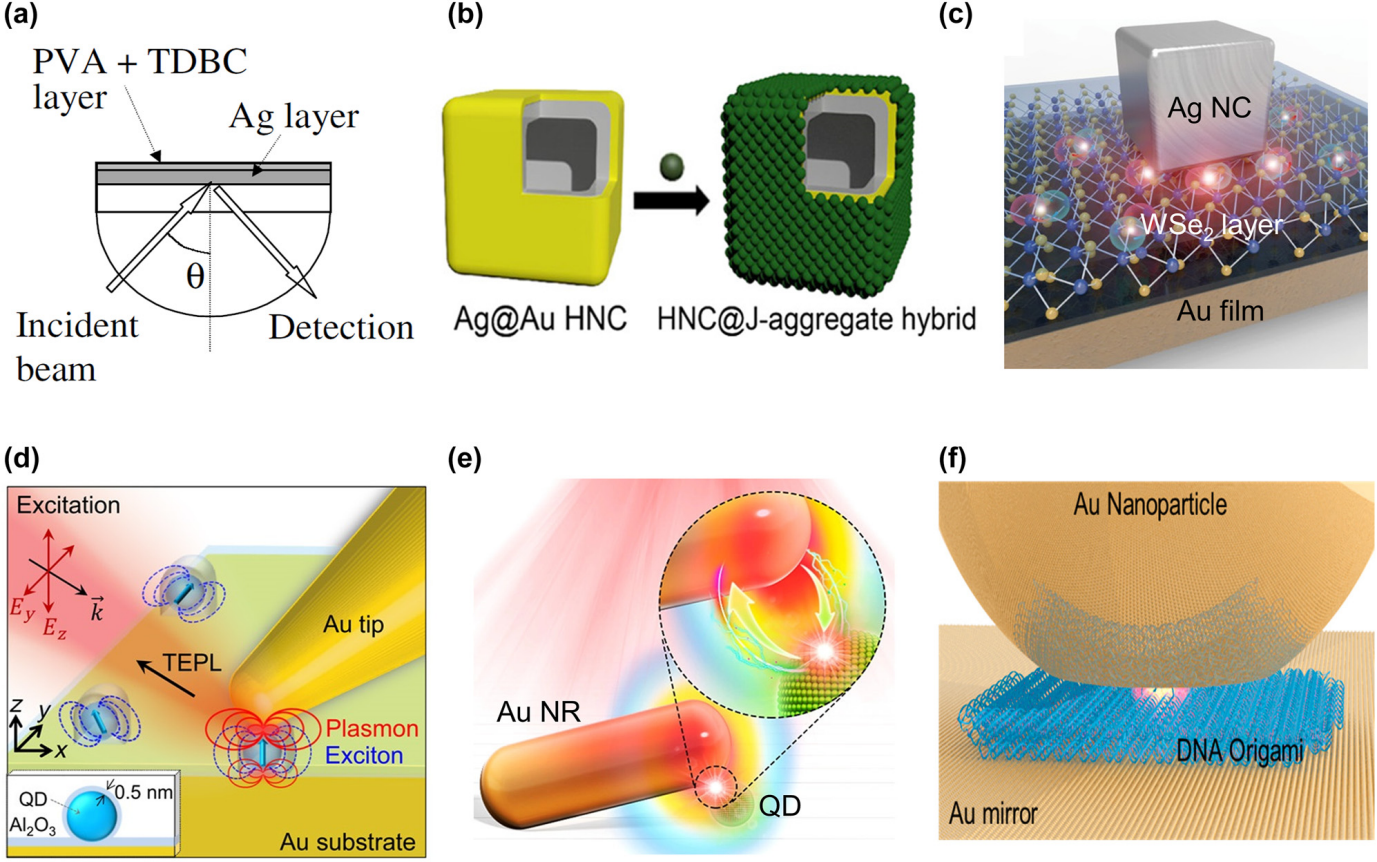
Diverse arrangements of nanostructures for plexcitonic strong coupling. (a) Ag film with 50 nm thickness and a TDBC J-aggregate layer on a hemispherical prism. (b) Ag@Au hollow nanocube coated with J-aggregates. (c) WSe_2_ monolayer between Ag nanocube covered PVP layer and Au film mirror. (d) QDs in the space between Au mirror substrate and Au tips with several nanometer apex radii. (e) Wedge nanogap composed of Au nanorod and QD on a carbon film substrate. (f) Using DNA origami, a single molecule is placed in the middle of the gap between the Au nanoparticle and the Au mirror. The pictures are reproduced with permission: (a) Ref. [41] © 2004 American physical society. (b) Ref. [80] © 2022 American chemical society. (c) Ref. [39] © 2018 American physical society. (d) Ref. [45] © 2019 American chemical society. (e) Ref. [121] © 2022 American chemical society. (f) Ref. [117] © 2018 American chemical society.

Plexcitonic strong coupling between SPPs and excitons was observed using a layered structure using metal films and exciton films [41, 97, 98]. This structure is on top of the hemisphere prism and strongly couples with SPPs and excitons. The reflection measurement showed Rabi splitting of 180 meV at room temperature [41] (Figure 2a). Biehs et al. [51] used hyperbolic metamaterials instead of simple metal films. The plasmonic nanostructures are designed so that holes or grooves are carved in the film to produce the effect of LSP in addition to SPPs in an array shape. The hole arrays [99, 100], nano-slit arrays [101], nano-disk arrays [102, 103], and gold nanoparticle crystals [50] have been used as array structures with high *Q*-factors or small mode volumes.

The interactions between excitons and LSP create strong coupling in systems including single plasmonic nanostructures. The most commonly used nanoparticles (NPs) of plasmonic nanostructures are nanoprisms [40, 104, 105], nanorods (NRs) [44, 106], nanocubes (NCs) [107, 108], and nanostar shapes [109]. In the interaction between silver nanoprisms and TDBC J-aggregates, 70 to 85 excitons contributed to the exchange, which showed the possibility of strong coupling with a single exciton [40]. Fofang et al. [110] developed nanoshell complexes in the shape of a sphere by coating an exciton layer on a metal nanoparticle with a core–shell. Nanoshells may also be integrated into other single nanostructures. Combined NR and nanoshells yield core–shell Au@Ag NRs [111], and an NC with nanoshells represents a cuboid Au@Ag NR [112]. Renming Liu et al. [112] succeeded in reaching strong coupling at the level of a single exciton and single plasmonic nanocavity with cuboid Au@Ag NRs and J-aggregate monolayers. Recently, nanoshells were designed as hollow nanocube (HNC) complexes, which were combined with nanocubes consisting of a Au layer, a Ag layer, and a J-aggregate layer [80, 113] (Figure 2b).

Plasmonic nanostructures have been developed in more complex configurations comprising coupled plasmonic nanoparticles to confine and enhance light at the nanometer scale promoting strong coupling occurrence. Dimer nanostructures may consist of a couple of nanospheres [114] or nanorods [115]. Dimer nanoprisms form a bowtie nanoantenna [79, 116]. Santhosh et al. [79] obtained a coupling rate of up to 120 meV for a single QD in a bowtie plasmonic nanostructure. Nanostructures consisting of metal films and nanoparticles are called a nanoparticle-on-mirror (NPoM) [7, 117, 118] if the shape of the particle is a nanosphere, and they are called nanocube-on-a-mirror (NCoM) [39, 119, 120] if the nanoparticles are nanocubes (Figure 2c). Chikkaraddy et al. [7] developed a system consisting of an NPoM with a 0.9 nm gap and a single methylene blue molecule. Chen et al. [119] fabricated NCoMs with a 3 nm gap. Instead of nanoparticles, Park et al. [45] applied Au nanotips to create a nanocavity (Figure 2d). Li et al. [121] made a wedge nano gap cavity using single nanoparticles with wedge-shaped complexes of gold nanorods and a single core–shell CdSe/ZnS QD and observed Rabi splitting of 234 meV (Figure 2e).

Placing excitons within very small nanoparticle hotspots, where interactions between the nanocavity and excitons are maximized, is a challenging problem. DNA-origami techniques render an elegant way to place excitons inside plasmonic nanostructure gaps. DNA templates are placed between two particles in the dimer resonant structures [40, 115], while they are placed between the NP and mirror in the NPoM [117, 118] to adjust the position of the excitons (Figure 2f).

### 3.2 Plexcitonic strong coupling characteristics using QD/J-aggregate

Quantum emitters (QEs) that include molecules, quantum dots, and single atoms support an exciton formation upon a photon absorption and, subsequently, exhibit a single photon emission [73]. An exciton is an electrically neutral quasi-particle formed by an electron–hole pair. Excitons are primarily composed of Frenkel excitons and Wannier–Mott excitons [122], which are distinguished by their differences in electron–hole binding energy and Bohr radius. Since Frenkel excitons (∼0.1–1 eV) have a higher binding energy than Wannier–Mott excitons (∼10–30 meV), Frenkel excitons are stable excitons, whereas Wannier–Mott excitons are unstable at room temperature [122, 123]. Frenkel excitons are mainly found in organic molecules such as J-aggregates, and Wannier–Mott excitons are formed in inorganic molecules such as GaAs quantum dot (QD) or MoS_2_ monolayers [23].

To unravel the characteristics of strong coupling, QDs and J-aggregates with well-known optical properties are typically employed as QEs. QDs possess a size of a few nanometers to several tens of nanometers and are also called artificial atoms [35]. QDs exhibit narrow emission bands, stable and bright PL, and easily controllable emission spectra via selecting the appropriate shape and size [35, 76]. Unlike fluorescent molecules, QDs are not prone to photobleaching against high-intensity excitation [76, 96]. On the other hand, J-aggregates are a type of molecular crystal dye with narrow absorption bands that are red shifted with increased sharpness compared to molecule monomers, which makes strong coupling easier to observe in these systems [76, 96].

**Figure 3:**
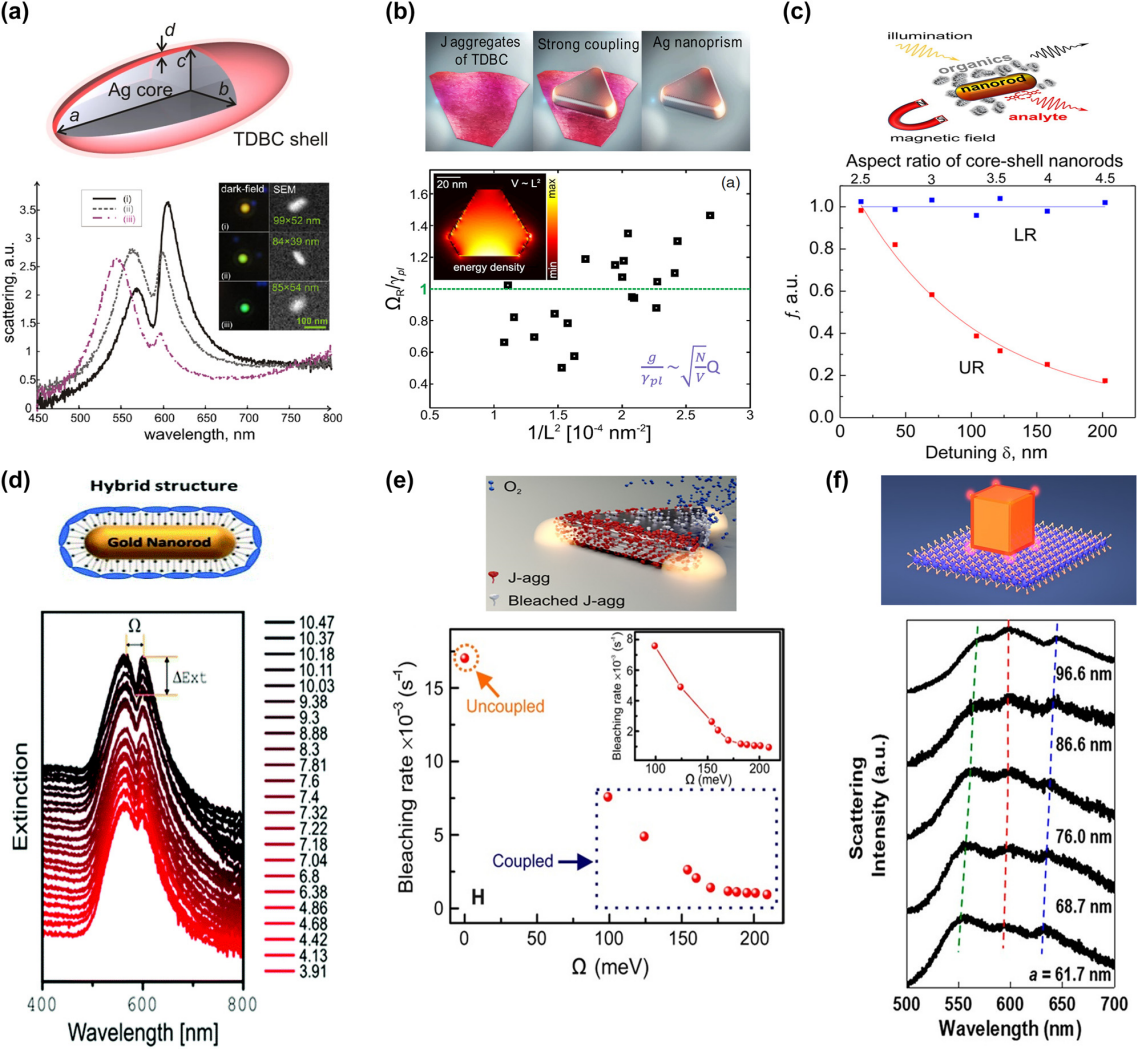
Physical and chemical characteristics of plexcitonic systems. (a) Scattering spectra of three individual plexcitonic systems with Ag nanorod and TDBC J-aggregate shell using the dark field. The inset contains SEM and dark-field images of the corresponding plexcitonic system. (b) Distribution of Ω_R_/*γ*_pl_ by the inverse function of geometric volume. The inset images are the energy density distribution of nanostructure. (c) The ratio spectra of normalized magnetic circular dichroism (MCD) signal hybrid nanorod/J-aggregates plexcitonic system and bare core nanorod. This spectrum shows the magneto-optical activity of upper resonance (red) and lower resonance (blue) for detuning (corresponding aspect ratio of nanorods). (d) Extinction spectra for a Au nanosphere and J-aggregate hybrid nanosystem in terms of PH value. (e) Bleaching rate of uncoupled J-aggregates and plexcitonic system with Ag nanoprisms and J-aggregates coupling. (f) Scattering spectra in which diexcitonic strong coupling is observed through the hybrid system with Au nanocubes and two exciton material J-aggregates and WS_2_. (a) Ref. [44] © 2013 the author(s). (b) Ref. [40] © 2015 American physical society. (c) Ref. [111] © 2017 American chemical society. (d) Ref. [124] © 2020 the royal society of chemistry. (e) Ref. [105] © 2018 the author(s). (f) Ref. [108] © 2021 the author(s).

Vasa et al. [101] utilized a system of a gold nanoslit array coated with a thin J-aggregated layer and observed reversible transient conversion from strong coupling to weak coupling on a sub-picosecond timescale. Zengin et al. [44] evidenced transparency dips, originating from strong coupling between a silver nanorod and TDBC J-aggregates. They also showed that the volume of nanoparticles affected the depth and width of this dip (Figure 3a). When a system with strong coupling involves a chiral plexcitonic system, the circular dichroism (CD) response accurately distinguishes the strong coupling regime [115]. Furthermore, Zengin et al. [40] demonstrated strong coupling between a single silver nanoprism and J-aggregates and noted that 2*g*/*γ*_pl_ and $Q/\sqrt{V_{m}}$ (mode volume *V*_*m*_, quality factor *Q*) are more important figures of merit for quantifying strong coupling at ambient conditions as compared to vacuum Rabi splitting (Figure 3b).

Bellessa et al. [41] observed luminescence band splitting of coupled TDBC J-aggregates and a silver layer on a hemispherical prism. The spectrum of the coupled system was shifted, and the linewidth was reduced compared to the original spectrum. A single nanoparticle plexciton formation has been observed in PL band splitting of J-aggregates, and the signal increased at extremely low temperatures [104]. The lifetime and PL intensity of the plasmon–exciton coupled states have been further studied using gold nanostars and J-aggregates [109]. Apart from Rabi splitting, plexcitons have shown optical nonlinearities at the femtosecond time scales upon direct laser excitation of the hybrid plexcitonic states [106, 110]. In addition, it was observed that the magneto-optical activity of the exciton was improved by a strong coupling [106, 111] (Figure 3c).

Plexcitonic strong coupling is resistant to environments such as temperature, pH, and intense light [124] (Figure 3d). However, strong coupling modifies the circumstances of photobleaching dynamics. Photobleaching probability is effectively adjusted depending on strong coupling and detuning, and hybrid states are more stable in this photochemical reaction [105] (Figure 3e). As a J-aggregate exciton number increases, the rising coupling strength results in a shorter lifetime of the hybrid upper band under upper band excitation when it becomes bleached because of the bottleneck effect [125].

Zhang et al. [108] showed the diexcitonic strong coupling system between two different excitons and plasmons in AuNCs. The AuNCs coated with a J-aggregate layer became AuNC@J-aggregate hybrid nanoparticles when the particles were placed on the WS_2_ monolayer. Plasmons in AuNC strongly interacted with Frenkel excitons in J-aggregates and Wannier–Mott excitons in WS_2_, and two plexcitons were observed in the scattering spectrum with two Rabi splittings resulting in three peaks [108] (Figure 3f).

## 4 Application of strong coupling in chemistry

Chemical reactions can be affected by the vibro-polaritonic strong coupling with vacuum fields or by the hot electrons produced from the plasmon–exciton coupling [126–128]. Strong coupling provides a tool to alter the vibrational energy states and rotational energy states or enhance energy and charge transport [22]. This section introduces vibro-polaritonic strong coupling, modal strong coupling, and strong plasmon–exciton coupling applications in chemistry that have been actively studied in recent years.

### 4.1 Vibro-polaritonic strong coupling in chemical reactions

**Figure 4:**
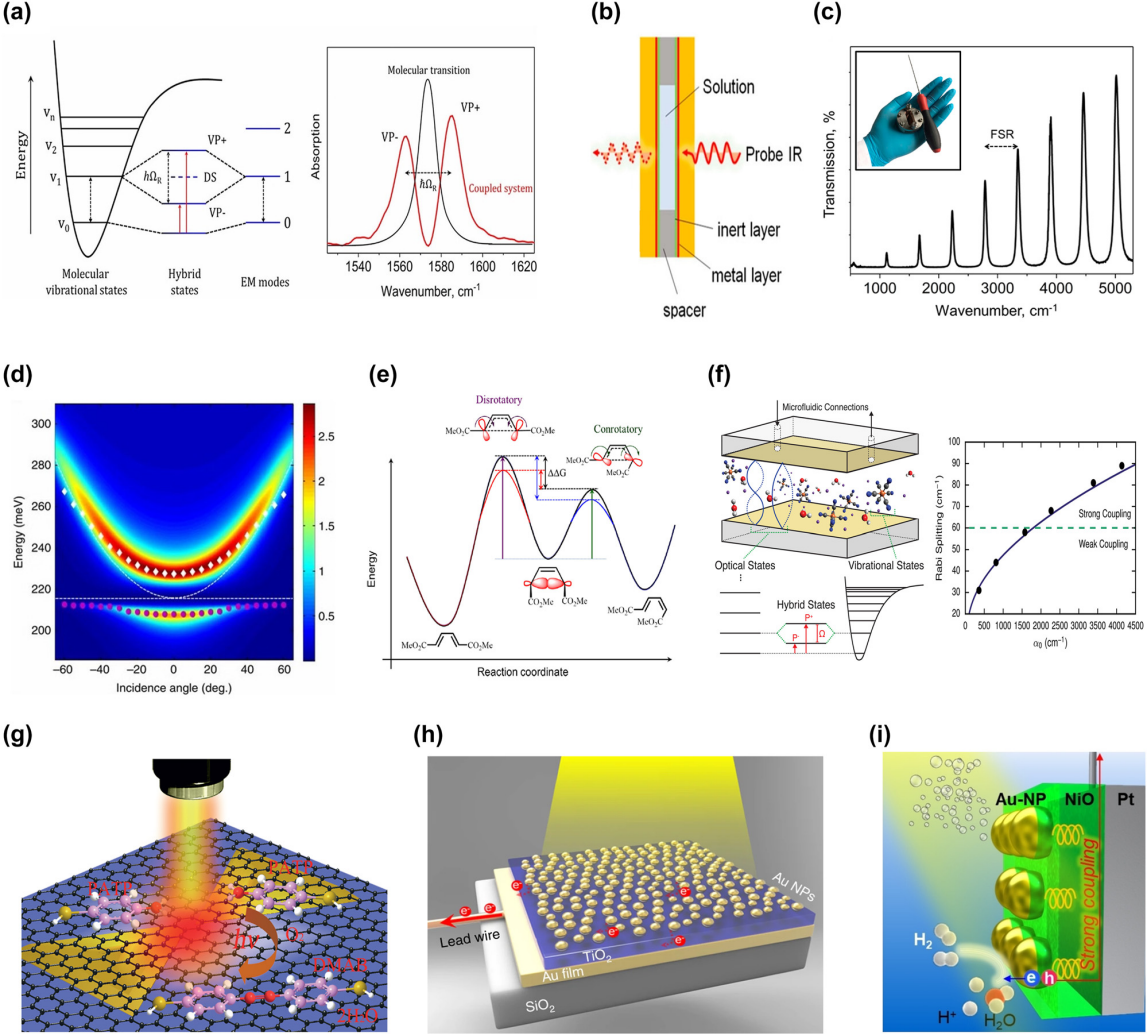
Modifying chemical reaction kinetics via coupling to cavity mode or plasmon mode. (a) Energy diagram (left) and Rabi splitting (right) of vibro-polaritonic strong coupling. (b) Schematic diagram of Fabry–Pérot microcavities. (c) Air-filled cavity resonance in FTIR transmission spectrum. The inset image is Fabry–Pérot microcavities and screwdriver to tune cavity length. (d) Optimal fitting calculation results from experimental-based data. Purple circles and white diamonds are real measured data. (e) Comparison of energy level diagrams of chemical reaction under noncavity or VSC mode. Gibbs free energy change (ΔΔ*G*) is modified by VSC to C=O (blue) and C–H (red). (f) Schematic showing the microfluidic cavity device and the energy state (left panel). Rabi splitting strengths for maximum absorption coefficient and boundary (green dot) between weak and strong coupling (right panel). (g) Strong plasmon–exciton coupling between Ag bowtie and graphene co-driven conversion reaction from PATP to DMAB. (h) Schematic of plasmon–cavity nanostructure that generates modal strong coupling. (i) Water reduction process using photocathode composed of ANP structure. The pictures are reproduced with permission: (a) Ref. [126] © 2021 American chemical society. (b) Ref. [131] © 2020 the author(s). (c) Ref. [126] © 2021 American chemical society. (d) Ref. [131] © 2015 the author(s). (e) Ref. [2] © 2020 the author(s). (f) Ref. [135] © 2016 American chemical society. (g) Ref. [145] © 2015 the author(s). (h) Ref. [156] © 2018 the author(s). (i) Ref. [162] © 2022 the author(s).

A method of changing the chemical landscapes through the hybridized light–matter states was introduced in 2012 by Hutchison et al. [3]. The authors showed that organic molecules of merocyanine yielded different photoisomerization rates when the electronic transition was resonantly coupled to a Fabry–Pérot cavity. Such a method has been applied to the vibration of molecules in the IR range as the resonant cavity length can exceed several micrometers facilitating experiments in the aqueous solutions [22, 126, 129]. The vibrational state of the molecule strongly interacts with the cavity mode and splits into two vibro-polaritonic states (Figure 4a). The cavity resonance can be fine-tuned to molecular vibrational transitions via control over the Fabry–Pérot microcavity length [130] (Figure 4b and c). For instance, carbonyl bond–stretching modes that exhibit transition energy around 215 meV and dictate the peptide bond formation can be strongly coupled to vacuum fields [131] (Figure 4d). Thomas et al. [132] reduced the ground-state deprotection reaction rate of PTA (1-phenyl-2-trime-thylislylacetylene) by 5.5 times through VSC between the vibrational state of Si–C and photons in cavity mode. In a chemical reaction under VSC, thermodynamic parameters can be modified in a dark environment and at room temperature. VSC may weaken the polarity of carbonyl moieties in aldehydes and ketones under the print cyclization process [133]. Among the aldehydes and ketones used in the print cyclization process, the reaction rate of four reactants, acetaldehyde, propionaldehyde, acetone, and cyclohexanone decreases under VSC. In contrast, the positive catalytic effect of hydrolysis of para-nitrophenyl acetate (PNPA) occurred through VSC of a C=O vibrational stretching state [134]. In the case of a compound capable of two chemical reactions, the corresponding reaction rates may be selectively controlled via VSC. In the chemical reaction of Si–C scission of *tert*-butyldimethyl((4-(trimethylsilyl)but-3-yn-1-yl)oxy)silane, the thermal dynamic parameters and ratio of the resulting two types of derivatives were changed under VSC to vibrational stretching modes of Si–C or Si–O bonds [83]. The keynote role of symmetry in chemistry under VSC was recently verified by altering the stereoselectivity of a pericyclic reaction that follows the Woodward–Hoffmann rules [2]. In the pericyclic response of cyclobutene, VSC to C–H bending modes affects the stereoselectivity of the chemical reaction and thermodynamic parameters and augments the amount of the symmetry-forbidden reaction product (Figure 4e).

Coupling strength, which changes the landscape of chemical reactions, depends on various factors. The coupling strength of the optical state and the vibrational state of Fe(CN)_6_^4−^ ions [135] gradually increases with the ion concentration, resulting in strong coupling at 15 mM (Figure 4f). In addition, the coupling strength of VSC rises with the concentration of the reactant, and this affects thermodynamic parameters [136]. The thickness of the FP cavity doesn’t affect the coupling strength [137]. Instead, the lifetime of photons in the cavity increases as the thickness increases, and the energy dissipation rate decreases. The coherence time of polaritonic states is enhanced by reducing the energy dissipation rate. Menghrajani and Barnes [138] focused on the plasmon coupling mode in microcavities. Such a two-mirror cavity couples to the coupling mode of the plasmon of the coated metal, which have the potential for modification of chemical reactions. Raman spectroscopy is a powerful tool in chemical analysis. Under VSC, Raman scattering shows the possibility of advanced tools such as surface-enhanced Raman scattering (SERS) and tip-enhanced Raman spectroscopy (TERS) based on Rabi splitting and amplifying the cross section [139].

### 4.2 Strong plasmon–exciton coupling co-driven catalytic reaction

SPR using metal nanoparticles causes effective catalyst reactions assisted with hot electrons [140]. Many studies have been conducted on applying SPs to surface catalytic reactions since 2010 [141, 142]. The surface chemical reactions using SPs have low efficiency and probability due to the low density and short lifetime of the hot electrons [143, 144]. To overcome this limitation, the coupling of plasmons and excitons that manifest a more efficient reaction has been studied [127].

The substances used for exciton generation are the graphene [145] (Figure 4g) and TiO_2_ [146]. At the same time, the catalysis of a chemical reaction is dictated by the number of layers of graphene and the presence of TiO_2_. Plasmon–exciton hybrid nanostructures of graphene–Ag nanowires are exploited as graphene-mediated SERS (G-SERS) substrates, increasing the efficiency and probability beyond the surface catalysis reaction of either the graphene or single Ag nanowires [147]. The graphene-based plasmon–exciton hybrid nanostructure shows significant advantages even in a liquid environment as a G-SERS substrate [148]. It also enables a changeable catalytic reaction with a gate and bias voltage [149]. The gate and bias voltages increase the DOS and higher energy of hot electrons, respectively, which increase the probability and efficiency of plexciton co-driven surface catalytic reactions. In addition, MoS_2_–Ag nanoparticle hybrid structures were developed with monolayers of MoS_2_ as an exciton-bearing material [150].

In these hybrid structures, the plasmon–exciton coupling depends on the size of the metal nanoparticles. The coupling strength increases with the overlap between the absorption peak of excitation and the resonance peak of the surface plasmon [151]. This strong plasmon–exciton coupling enhances the Fermi level of a plexciton hybrid system [152], which ameliorates the efficiency of surface catalytic reactions. Whereas hot electrons and holes are essential entities involved in the mechanisms of these systems [153]. Hot electrons and holes dictate the flow of surface catalytic reactions. Experimentally, it has been demonstrated that hot electrons drive reduction reactions, while hot holes promote oxidation reactions [154]. The plasmon–exciton hybrid structure can be employed in the field of SERS, TERS, or surface catalytic reactions [4, 155].

### 4.3 Modal strong coupling in photoelectrochemistry

Strong coupling in systems where two resonant modes are coupled is attributed to modal strong coupling. While plexcitons and vibro-polaritons arise from strong coupling between a cavity mode and an emitter, the modal strong coupling is formed by strong interaction between plasmon mode and cavity mode without an emitter. Recently, studies on modal strong coupling between a plasmon mode (metal nanoparticles) of LSPR and a cavity mode of Fabry–Pérot nanocavity (TiO_2_/metal film) were reported in the field of photoelectrochemistry [128]. Shi et al. [156] exploited Au-NP/TiO_2_/Au-film (ATA) structures for intensifying water splitting under modal strong coupling conditions. The authors illustrated that the strength of the coupling varied depending on the depth of inlay for Au NPs and the thickness of the TiO_2_ layer (Figure 4h). The ATA structure undergoes high absorption of light, improves internal quantum efficiency (IQE), and can function as a photoanode in the water splitting reaction [156] and ammonia photosynthesis system [157]. The modal strong coupling within oxygen evolution reactions (OERs) promotes the generation of electron–hole pairs; as a result, a positive Fermi level shift is observed through graphene-based electrochemical surface-enhanced Raman spectroscopy measurements [158]. Triethanolamine contributes to hot electron generation and transfer processes as an effective electron donor, helping to grow incident photon-to-current conversion efficiency (IPCE) and IQE [159]. Upgraded ATA structures, *e.g.*, postdeposited Au on an ATA (Au@ATA) and Au–Ag alloy NP/TiO_2_/Au films (AATA), have been developed to increase IPCE and IQE efficiency [160, 161]. In addition, a new Au NP/p-type NiO/Pt film (ANP) structure was used as a photocathode to boost a hot-hole transfer to a NiO semiconductor and accelerate water reduction by hot electrons [162] (Figure 4i).

## 5 Strong coupling for detecting and analyzing biomolecules

**Figure 5:**
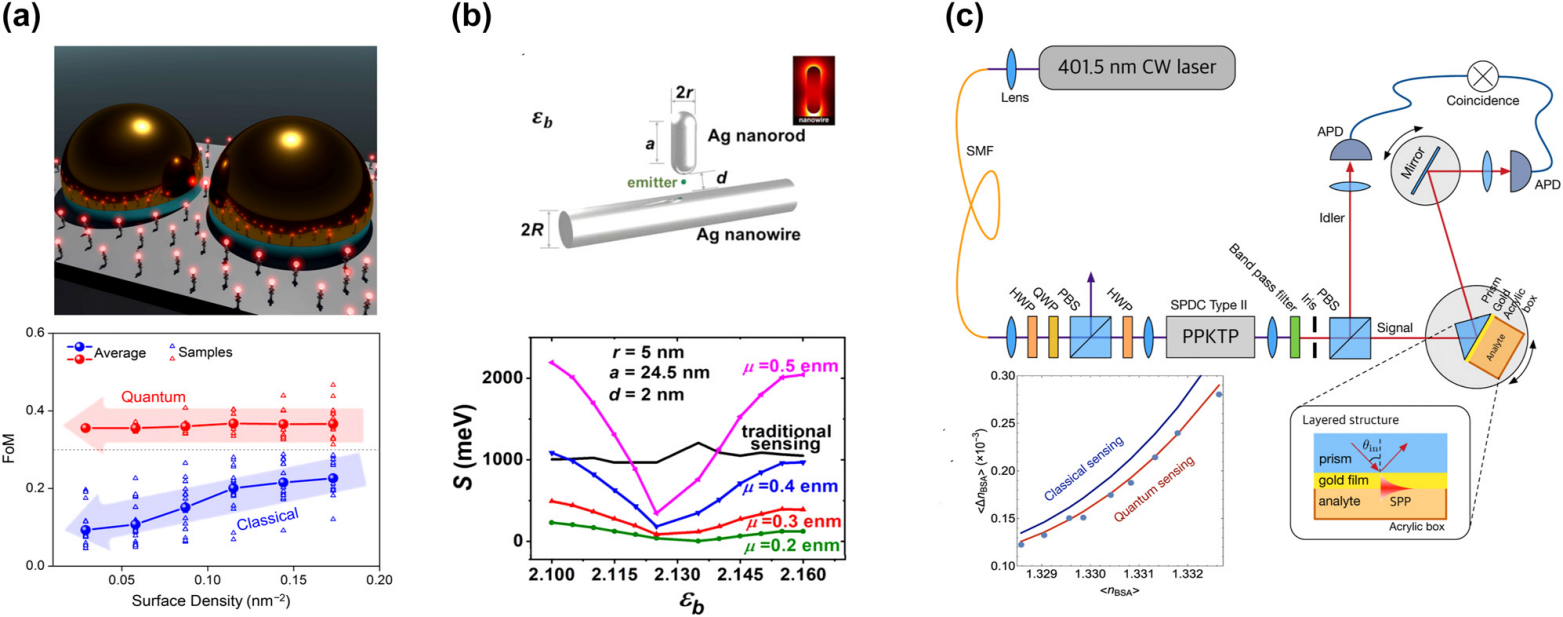
Plexcitonic and quantum-enhanced biomolecular sensors. (a) Strong coupling–assisted immunoassay sensing comprises a plasmonic dimer nanoantenna, an antigen, a sensing label, and two antibodies. The panel below illustrates the sensitivity figure-of-merit as a function of molecule density in the hotspot. (b) Theoretical demonstration of Ag nanorod–Ag nanowire sensing performance in the strong coupling regime. The panel below shows the sensitivity of such a nanocavity biosensor with a 2 nm gap size as a function of the emitter permittivity and transition dipole moment. The black curve corresponds to traditional plasmon sensing. (c) Quantum-enhanced Kretschmann SPR sensor setup. The panel below depicts the reduction of the refractive index uncertainty measured in the quantum sensing configuration (heralded photons) compared to a classical SPR sensor. The pictures are reproduced with permission: (a) Ref. [175] © 2019 American chemical society. (b) Ref. [176] © 2020 IOP publishing ltd. (c) Ref. [181] © 2018 optical society.

Strong cavity–emitter interaction may enable probing quantum coherence in biological systems [94, 96, 163, 164]. For instance, photosynthesis begins with rapid and efficient energy transfer between pigments of the light-harvesting complex and its reaction center [165–168]. Long-lived quantum coherence supports practically lossless energy transport toward the target. Generation of hybrid cavity–biomolecule states in the strong coupling regime is a possible approach to mimic and understand the processes in photosynthesis at the quantum level [169, 170]. Moreover, strong coupling–based biosensors may improve their sensitivity beyond the limit of classical sensors [163, 171]. In addition, a strong coupling can occur between a cavity and energy levels of chlorosomes inside a living cell at ambient conditions [9, 172]. In this section, we highlight recent advances in the applications of strong-coupling phenomena for biosensing, generation of a “living polariton,” and probing plasmon–exciton hybrid states of absorption bands of light-harvesting complexes and vibrational states of proteins. These works may open a route toward understanding quantum biological processes and creating next-generation biosensors (Figure 5a).

### 5.1 Sensing applications

#### 5.1.1 Plexcitonic sensors

Plasmonic nanostructures are intensively employed for label-free biosensing applications [10] due to their ability to confine light in sub-diffraction volumes, increasing the sensitivity of the LSPR to the refractive index of the nanoenvironment in the cavity hot spot. Integration of plasmonic nanocavities or hybrid plasmonic/whispering-gallery-mode microcavities enables nonfluorescent and label-free single-molecule sensors of biomolecules with molecular weight down to 1 kDa [173]. Nevertheless, the classical plasmonic sensors are limited by quantum shot noise and feature low selectivity. They cannot probe the quantum effects in biomolecules or living cells as the plasmon and exciton modes remain uncoupled [6, 170]. A strong coupling regime may provide a sensitive platform for sensing various types of biomolecules as any slight change in the cavity environment perturbs its optical modes [174]. Kongsuwan et al. [175] proposed gold dimer antennas with gap sizes less than 5 nm for quantum immunoassay sensing (Figure 5a). They demonstrated plexcitonic strong coupling with a label molecule attached to an antigen when the latter binds to an antibody in the gap. The authors observed a 15-fold sensitivity improvement over the traditional plasmonic sensors whose sensitivity is entirely based on the LSPR wavelength shift per refractive index unit (RIU) [10, 94]. This improvement primarily originates from Rabi splitting of the energy levels, which is equivalent to a bidirectional plasmon resonance shift. Moreover, the authors developed a figure of merit for their sensor that does not decrease with the number of coupled molecules indicating its quantum nature. This approach may provide sensitivity down to a single QE. Qian et al. [176] predicted that silver nanorod/silver nanowire cavities offer a platform for efficient biosensors in the strong coupling regime, whose sensitivity is deduced as the coupling strength change per refractive index unit (RIU). The sensitivity of these sensors also may outperform traditional plasmonic sensors by more than an order of magnitude if the nanocavity gaps are extremely small (1–3 nm) and the significant electric dipole moments of the emitter are maintained (Figure 5b).

Dutta-Gupta et al. [177] reported a theoretical work regarding strong coupling between an SPP mode of silver film and hemoglobin energy states (*Q*-bands). The first electronic transition in hemoglobin produces the *Q*-bands, while their spectral position is related to the hemoglobin deoxygenation [178]. The sensitivity of the oxidized hemoglobin detection increases up to 10 times for the coupled system compared to the decoupled case (conventional plasmonic sensor) due to the bidirectional Rabi splitting of *Q*-bands of hemoglobin absorption spectra. This strong coupling interaction could be of interest for oxygen sensing in human blood.

Roxby et al. [179] demonstrated a sensor of heavy ions based on photocurrents of microalgae enhanced by strong coupling. Photoelectrical efficiency can be significantly amplified when the photosynthetic fluorescence of microalgae is excited in the vicinity of copper NPoM cavities. Based on this amplification, the perturbation of microalgae-induced photocurrent in the presence of nanomolar concentrations of cadmium, iron, chromium, and manganese ions becomes perceivable within several seconds after injection.

#### 5.1.2 Quantum plasmonic biosensors

Integrating quantum plasmonic sensors may circumvent the shot-noise limit by taking advantage of the quantum input states and measurements [180]. For instance, a quantum-enhanced sensor in the Kretschmann configuration can reduce its noise floor beyond the shot noise limit via quantum squeezing. Lee et al. [181] showed an experimental setup where the presence of a single photon that underwent the interaction with the sensor was heralded by the detection of its twin photon in the idler channel. The authors were able to detect biomolecules such as bovine serum albumin. The uncertainty of the measurement manifested a ∼20% reduction for the quantum biosensor compared to the classical one, whereas the quantum enhancement was given by the total transmission of the system (Figure 5c). Sadeghi et al. [182] reported a theoretical quantum plasmonic sensor based on quantum dot coupling with an Ag nanorod, which was investigated in the presence of ultrafast polarization dephasing of the quantum dot. AgNR–QDs structures were designed so that quantum coherence effects were still present at ambient conditions. The nanorod functionalized with receptors of target biomolecules yielded a minimal Förster resonance energy transfer (FRET) with the quantum dot. At the same time, the coherent blockage of FRET was lifted upon target biomolecule adsorption, which opened another nonradiative energy loss pathway and, consequently, quenched the QD photoluminescence. The coherent plasmonic field in quantum dot–nanoantenna hybrids may enable the study of position-dependent dynamics in the vicinity of the nanoantennas [183], as the specific field properties are unique at each point. Quantum dots attached to a gold nanorod sensor may probe the field time delay in the quantum dots via coherent plasmonic effects, yielding resolution down to 10^−6^ RIU [184].

### 5.2 Strong coupling with biological molecules

#### 5.2.1 Light-harvesting complexes

**Figure 6:**
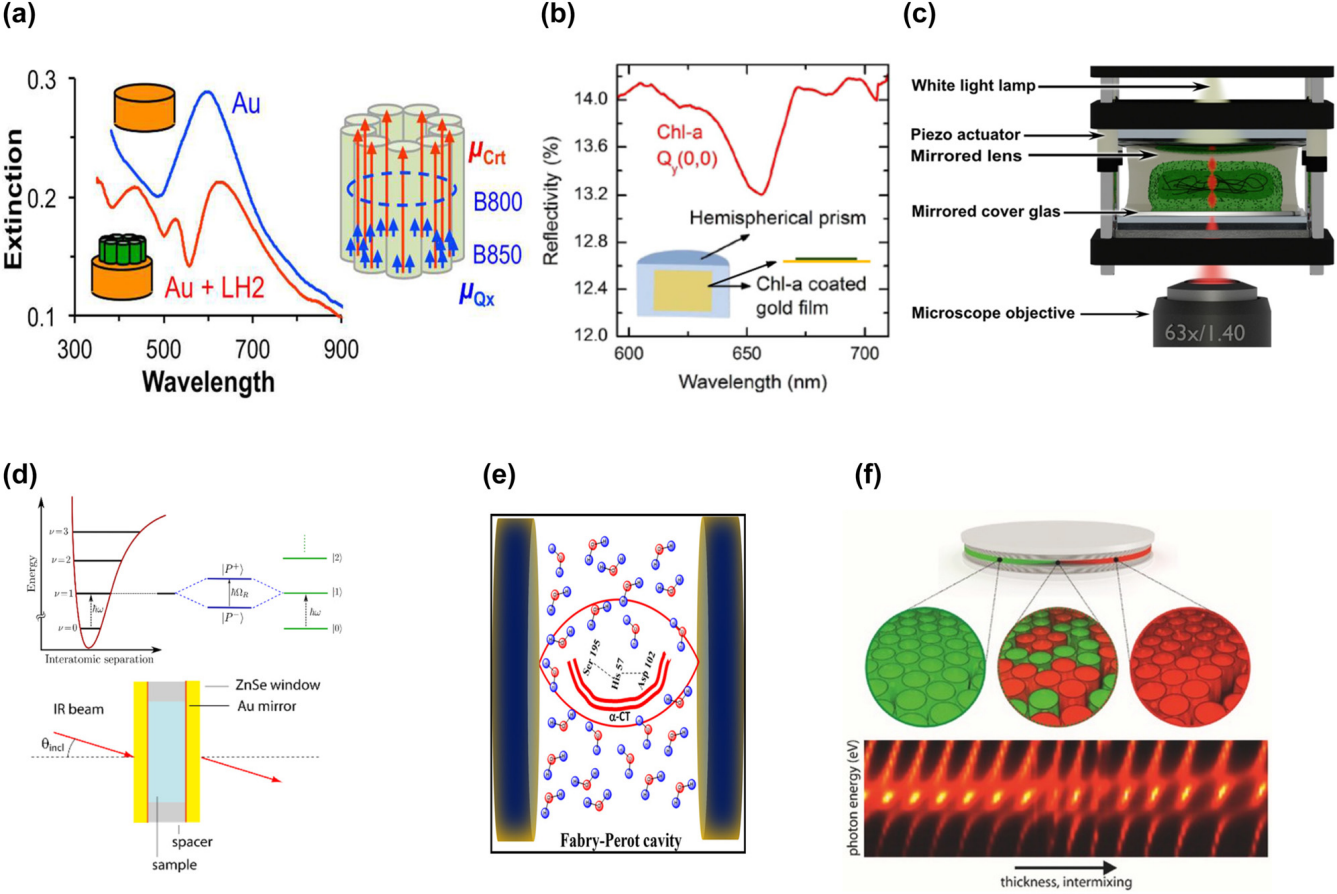
Applications of strong coupling in biophysics. (a) Extinction spectra for gold nanostructure (blue) and with LH2 (red), and schematic illustration of his-tagged LH2 with Crt and B850 *Q*_*x*_ transition dipoles. (b) Schematic diagram showing hybrid nanostructure consisting of Chl-a and gold film on a hemispherical prism. At 656 nm, there is a dip in the reflectivity of Chl-a molecules. (c) Fabry–Pérot microresonator setup with cavity including two mirrors, piezoelectric actuators, and bacteria. (d) Schematic diagrams of energy level states for VSC with a protein (top) and experimental setup (bottom). (e) Sketch of Fabry–Pérot cavity resonator used to study the catalytic activity of enzyme α-CT under VSC of water. (f) Strong coupling scheme between a Fabry–Pérot cavity and fluorescent proteins (tdTomato and eGFP). The pictures are reproduced with permission: (a) Ref. [191] © 2016 American chemical society. (b) Ref. [169] © 2019 American chemical society. (c) Ref. [189] © 2021 the authors. (d) Ref. [86] © 2016 American chemical society. (e) Ref. [198] © 2020 American chemical society. (f) Ref. [199] © 2016 the authors.

The first step of the photosynthesis process is associated with an ultrafast and efficient energy transfer within the light-harvesting complexes benefitting from the quantum coherence [185, 186]. The light-harvesting complexes exhibit single-photon emission at ambient conditions, where all chromophores inside it are efficiently coupled with the reaction center [187]. The exciton transport through a chain of two-level systems becomes significantly enhanced under the strong coupling regime, as the transport occurs via delocalized polariton modes rather than via tunneling processes between two-level systems [9, 188]. Given this circumstance, the strong coupling of light-harvesting complexes may suggest a method to study the quantum coherence involved in the energy transfer between pigments [189]. The strong coupling of light-harvesting complexes with the microcavities [6, 172, 189, 190] and plasmonic nanostructures [191, 192] has been the subject of extensive study in the recent years. Tsargorodska et al. [191] employed gold nanodisk arrays to probe strong coupling with light-harvesting complexes LH1 and LH2 as well as with maquette proteins and isolated *bacteriochlorophyll a* (*BChl a*) molecules. The authors claim that the strong coupling dramatically depends on the organization and coupling of the chromophores in the protein structure, which might play a significant role in observed coupling strength. For instance, the LH1 carotenoid-free complexes and maquette proteins containing chlorine as a pigment yielded different coupling strengths with the nanodisks arrays, indicating that the effective electric dipoles differed for those proteins. Furthermore, light-harvesting complexes without carotenoids, in which *BChl a* complexes are the only absorbers [193], yielded Rabi energy splitting of 100 meV, whereas energy splitting for a monolayer of independent *BChl a* molecules was not observed. These findings indicate that a particular presentation of absorbers in light-harvesting complexes is crucial for a strong coupling regime (Figure 6a).

An NPoM cavity consisting of a silver nanocube on top of a thin gold film is an excellent candidate to generate plexcitonic states with biomolecules, as the cavity nanogap size can be controlled with a sub-nanometer precision [19]. Yuan et al. [170] reported strong coupling between this NPoM and the chlorophyll a (Chl a) complexes with a maximum Rabi splitting energy of 120 meV. The energy splitting of the hybrid modes was high enough to observe strong coupling in a spectrometer-free configuration, allowing the authors to determine the coupling strength in real-time based on the RGB values of the dark field image. When the coupling strength of a cavity to Chl a reached 90 meV, the radiative rate enhancement started to gradually fall at higher coupling strength, which was interpreted as an effect reminiscent of Purcell breakdown. Singh et al. [169] demonstrated a strong coupling of propagating SPPs of a gold thin film and localized plasmons in gold nanoelipsoids to excitons of Chl a (Figure 6b). Similar to [170], the observed photoluminescence of Chl a was quenched by strong coupling with localized plasmons associated with ultrafast relaxation dynamics. Thanks to the presence of a capping layer and dilution control experiments, the authors ruled out unwanted energy transfer to metal that typically causes a PL quenching [194, 195]. This experiment proposes an approach to mimic the oscillatory population dynamics [168, 196] and luminescence suppression occurring in photosynthesis. Ding et al. have leveraged silver nanoparticles as plasmonic nanocavities with Chl a molecules to enter the strong coupling regime [192]. Taking advantage of enhanced photon and phonon absorption via ultrafast plasmon–exciton exchange in Ag/chlorophyll *a* hybrid, the authors observed an increase in upconversion fluorescence by more than one order of magnitude.

#### 5.2.2 Living polariton

Coles et al. have reported the strong coupling between chlorosomes of a living cyanobacterium *Chlorobaculum tepidum* and a Fabry–Pérot microcavity with semitransparent mirrors [6]. Rabi energy splitting of 103 meV was observed while the cyanobacteria were still alive, confirming the generation of a “living polariton,” *i.e.*, a photon–polariton hybrid inside a living organism. Moreover, strong coupling between Chl a pigments in cyanobacteria *S. elongatus* and a Fabry–Pérot microcavity has recently been demonstrated [189] (Figure 6c). The photobleaching of light-harvesting complexes in the cyanobacteria gradually reduced the number of excitons involved in the coupling. The Rabi splitting monitoring allowed the authors to determine the number of active light-harvesting complexes in real-time. Strong coupling within a living organism could enhance energy transfer to the chlorosome reaction centers in living cells and light-harvesting ability. Understanding whether the light-harvesting complex energy tuning affects bacterial growth could be uniquely acquired from experiments in the strong coupling regime [172]. Altogether, studies on whether certain bacteria could adapt better to live in superposition with cavity modes would be of interest in the field of optics and quantum biology.

#### 5.2.3 Enzyme structure perturbation and activity control

Light-harvesting complexes feature absorption bands in the visible or infrared range [6, 165, 191]. In contrast, the specific vibrational states of various individual proteins could be coupled to cavity modes in the mid-infrared spectral range. Vergauwe et al. [86] demonstrated a strong coupling of vibrational states of amide-I’ in bovine serum albumin (BSA) with the vacuum field of Fabry–Pérot microcavities (Figure 6d). The observed vacuum Rabi splitting (67.1 cm^−1^) exceeds the sum of the cavity and vibrational mode linewidths. The selective VSC of protein bonds may enable tunable perturbation of the protein structure that is often required for investigating the mechanism of protein function. Later, the same group demonstrated the effect of VSC of water OH-stretching modes on the enzyme activity [197]. Water molecules are crucial for maintaining correct enzymatic reaction kinetics and are present in sufficient amounts for practical strong coupling experiments in aqueous solutions. The authors found that the proteolytic activity of pepsin can be reduced by 4.5 times when the OH-stretching modes are strongly coupled with a cavity. Lather and George [198] showed that VSC of OH-stretching modes increases α-chymotrypsin activity due to the cooperative effect (Figure 6e). Altogether, VSC offers new ways to alter enzyme reactivity or modulate the globular structure of proteins.

#### 5.2.4 Strong coupling with fluorescent proteins

Fluorescent proteins (FPs) exhibit a unique characteristic to form a visible-wavelength chromophore within its sequence. Enhanced green fluorescent protein (eGFP) and tdTomato exhibit high fluorescence brightness and photostability [199]. Dietrich et al. [199] showed strong coupling between Frenkel excitons of these FPs and a Fabry–Pérot cavity. The Rabi energy splitting can be controlled by adjusting the mixing ratio of eGFP/tdTomato blend deposited as a thin film inside the cavity (Figure 6f). Moreover, the photonic and excitonic components of polariton were tuned by the cavity length gradient established by tilting one of the cavity mirrors and the mixing ratio gradient of protein blends, respectively. Additionally, the same research group [200] demonstrated lasing capability of the microcavity with eGFP, which is promoted by the reduced exciton–exciton annihilation between large eGFP molecules and low photobleaching probability. The authors observed polaritonic lasing at low excitation energy (∼12 nJ), blue-shifted, concerning the eGFP gain spectrum. The second threshold evidenced photon lasing appearing at pump energy above 125 nJ. Photon lasing occurred at higher pump energy when the ground state of the eGFP turned out to be fully depleted and the system left the strong coupling regime. These studies revealed the effectiveness of fluorescent proteins as optical materials and paved the way for controlled manipulation of energy transfer and macroscopic quantum effects in ensembles of fluorescent proteins.

## 6 Outlook for strong light–matter coupling applications in quantum biology, life science, and biomedical engineering

**Figure 7:**
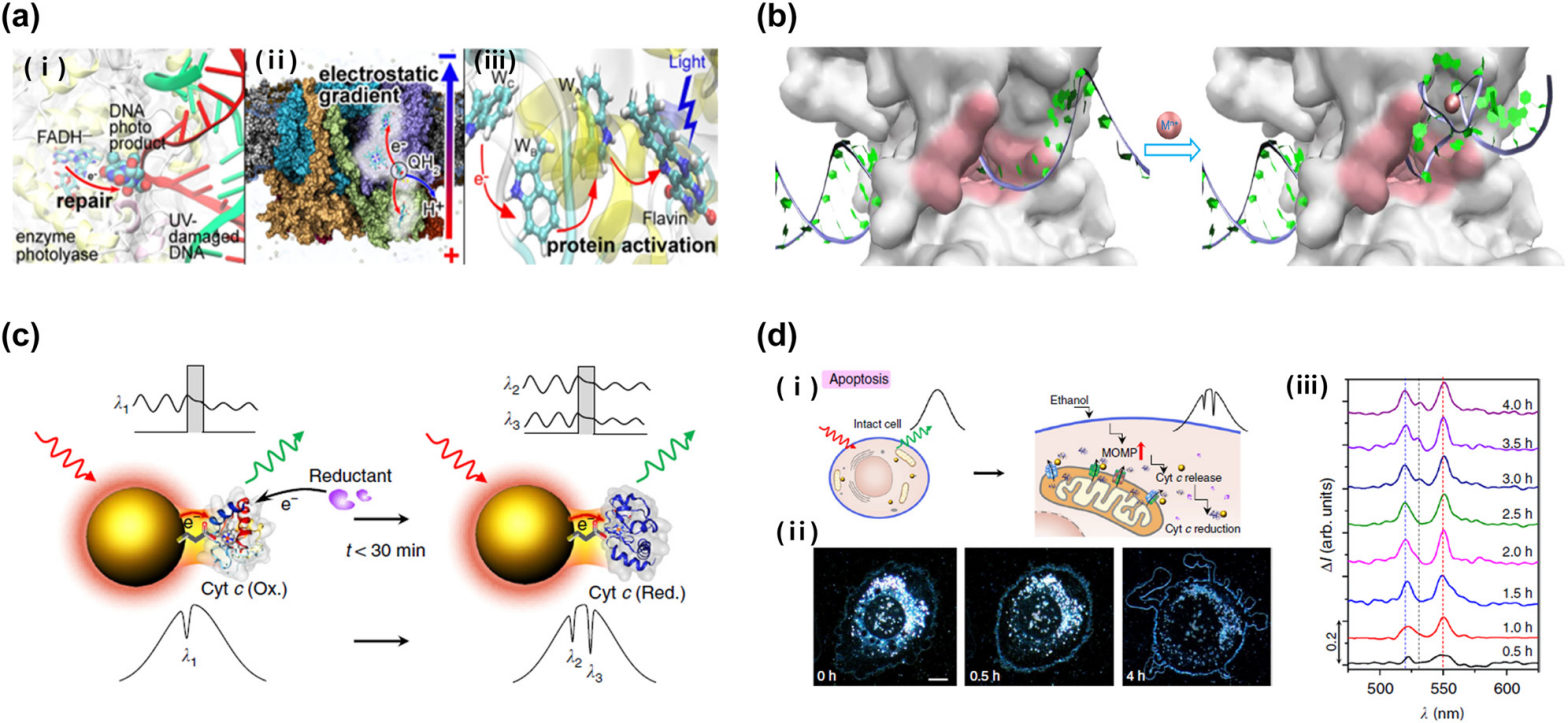
Outlook for strong light–matter coupling applications in quantum biological electron transfer. (a) Biological processes relying on electron transfer: (i) electron transfer in DNA repair by enzyme photolyase. (ii) Electrostatic gradient through the plasma membrane by electron transfer. (iii) Activation of protein by blue light forms a radical pair. (b) Schematic illustration of conformation between ssDNA and metal ions. Electron transfer between DNA and metal ions affects the gyration radius of ssDNA, so PCR efficiency decreases. (c) Schematic image of QBET imaging during Cyt c reduction in a tunnel junction. (d) The process of real-time sensing of Cyt c reduction during apoptosis. (i) Schematic image of apoptosis. (ii) Dark field image of the cell at the beginning of apoptosis measurement. (iii) Difference between scattering spectra of apoptosis showing QBET tunnel junction at different measuring times. The pictures are reproduced with permission: (a) Ref. [185] © 2015, the author(s). (b) Ref. [208] © 2022 American chemical society. (c) Ref. [204] © 2019, the author(s). (d) Ref. [204] © 2019 the author(s).

Mechanisms of various biological processes rely on electron transfer. Electron transfer in natural environments is systemically characterized in the formalism of quantum theory to describe its theoretical role in metabolic pathways. For instance, the magneto-reception of birds stems from spin-correlation via the light-assisted formation of radical ion pairs [201, 202]. Humans could recognize odor thanks to the electron movement coupling to the vibrational excitation of odorant [203]. In the presence of a potential barrier between the electron donor and acceptor, the electron transfer undergoes via quantum tunneling, which is conventionally called quantum biological electron transfer (QBET) [204]. Furthermore, electron transfer drives biological processes related to the cell life cycle, such as DNA repair (Figure 7a), DNA replication (Figure 7b), mitochondrial respiratory functions (Figure 7c), cell apoptosis (Figure 7d), and cellular homeostasis. These processes are gradually understood thanks to the advancement of quantum biology and biomedical applications [204].

Nevertheless, direct characterization of electron transfer on the atomic level under a controlled environment remains a challenging task in experimental studies. As discussed through this review, the coherent quantum dynamics processes become observable and enhanced under the strong coupling regime. Therefore, the strong coupling systems are expected to bring new insights to the current quantum biology studies, yielding enhancement of electron transfer [69], or extension of energy transfers within biological processes [188]. Fabry–Pérot cavities may enable the studies of strong coupling inside a living cell, thanks to the large mode volume. In contrast, plexcitonic nanocavity systems can be leveraged for local single atom–single photon couplings. In this chapter, we discuss relevant quantum biological processes related to cell life that could be potentially studied and controlled under strong coupling opening new avenues to biomedical applications.

### 6.1 Selective DNA replication by QBET

DNA replication is a vital process in the cell division of all living creatures that nearly perfectly copies the genome’s DNA. Complementary strands are synthesized by pairing nucleotides to a parental template single-stranded DNA (ssDNA) as DNA polymerase moves along the DNA helix [205]. Metal cations have been shown to affect DNA replication as they can strongly interact with negatively charged DNA [206]. The multivalent metal cations tend to induce condensation of polynucleotide chains, nonspecific aggregation, or helix-to-coil transitions [207]. Recently, evidence of QBET between DNA and metal ions has been reported [208]. The QBET influence on the DNA replication process has been theoretically investigated using molecular dynamics and quantum chemical calculations [208]. When the QBET occurs between DNA and metal ions, it creates an entangled conformation of single-strand DNA (ssDNA) and metal ions (Figure 7b). The concentration and valence of metal ions affect the conformation of ssDNA and, consequently, the efficiency of DNA replication. Moreover, metal ion binding to ssDNA can block DNA polymerase nanopores. Benefitting from intrinsic electrophilicity (active valence shell), metal ions can affect QBET by occupying protein binding sites [209]. Particularly, metal cations of high valence, *e.g.*, Au^+3^, Cr^+3^, tend to yield higher binding energy and QBET interaction with remote nucleotides. Based on the binding energy and net charge per base, the authors evaluated the gyration radius of ssDNA, *i.e.*, a metric that quantifies the impact on DNA conformation for various ions [208].

DNA replication mechanism is widely exploited for *in vitro* biomedical applications including polymerase chain reaction (PCR) and DNA sequencing. Selective PCR of DNA amplicons with different base pair numbers can be conducted in the presence of different concentrations of metal ions [208]. The DNA gyration radius reduction directly correlates with PCR efficiency. Due to metal ion impact on the DNA conformation, the DNA replication results in a low yield of long amplicons at high ion concentrations validated by electrophoresis. Consequently, PCR systems amplifying only short DNA sequences could be designed via a correct combination of the ion valences and concentration windows paving the way for multiplexed selective PCR. Creating multiplexed selective PCR amplifications on chips by quantum nanophotonics can provide an innovative solution for preventive medicine.

### 6.2 Cell respiration

Cellular respiration is the oxidative phosphorylation or respiratory chain–linked adenosine triphosphate (ATP) synthesis from adenosine-diphosphate (ADP) and inorganic phosphates in archaea, bacteria, and eukaryotes [210]. The underlying mechanism implies coupling respiratory chain electron transfer with final ATP synthesis. In eukaryotes, protein complexes I, III, and IV embedded in an inner mitochondrial membrane translocate protons through the membrane within the respiratory chain assisted by a series of electron transfers. According to chemiosmotic theory, redox reactions of the respiratory chain create proton-motive force across the inner mitochondrial membrane, which enables transmembrane proton movements for eventual ATP synthesis. The proton translocations through the membrane are catalyzed by electron transfers in the opposite direction [210]. Complex I supports the electron tunneling process between nicotinamide adenine dinucleotide (NADH) and ubiquinone [210]. Complex III (cytochrome bc_1_) carries two electrons to cytochrome c/cytochrome c oxidase and the diheme b-type cytochrome, respectively. Complex IV (cytochrome c oxidase) promotes the catalysis of electron transfer to molecular oxygen. The oxygen content in the cellular microenvironments must be sufficient for a thermodynamically favorable oxidative phosphorylation [211]. In contrast, around 2% of consumed oxygen within the scope of cellular respiration may be converted to reactive oxygen species (ROS) [212, 213]. Finally, an ATP synthase of animal mitochondria yields an ATP molecule per 2.67 translocated protons [210, 214].

Inhibition of electron transfer from NADH to oxygen within the respiratory chain transfer would yield insufficient oxygen consumption and ATP production by mitochondria [215]. Therefore, the dysfunctional electron transport chain (ETC) results in defective mitochondrial functions and diseases [213]. Redox-active molecules with modest redox, *e.g.*, methylene blue, can reciprocate the electrons in biological systems, improving metabolic activity and suppressing ROS formation by mitochondria [216]. Alternatively, the inner-membrane–bound gold nanoparticles (GNPs) have been shown to boost the ETC activity of dysfunctional mitochondria by efficiently mediating electron transfer with Cyt c [213]. Thanks to the presence of Cyt c absorption bands in the visible range, binding of GNPs to the inner membrane can be experimentally validated by plasmon resonance energy transfer (PRET) with oxidized or reduced Cyt c in the respiratory chain. Moreover, the reduced Cyt c absorption spectrum splits into two absorption bands; as a result, the presence of reduced Cyt c can be likewise captured from the corresponding spectral locations of the PRET-induced scattering quenching dips (Figure 7c). Enhanced oxygen consumption rate and ATP yield evidence of the effectiveness of the approach. Therefore, precise controls of ETC via light-molecular modulation can provide possible solutions for transformative medicine. Altogether, understanding mitochondrial ETC within the natural nanocavity formed between the inner and outer membranes of mitochondria benefits discovering the pathogenesis of neurodegenerative diseases and cancer.

### 6.3 Mitochondrial apoptosis signaling by electron transfer

Apoptosis is the best-known form of programmed cell death and is essential for maintaining the tissue homeostasis, embryonic development, and correct immune system functioning [217, 218]. The apoptotic process can be launched by radiation, heat, hypoxia, or specific drugs [218]. Deregulation of apoptosis results in degenerative diseases, autoimmune diseases, and cancer. Mitochondria are strongly related to cell death signaling because they contain proapoptotic factors such as Cyt c, endoG, and smac/diablo that trigger cell death pathways upon release in the cell cytosol [219, 220]. They initiate enzymatic reactions that lead to the degradation of DNA and proteins by secretion of cytosol during apoptosis [220]. In the frame of the mitochondrial pathway of apoptosis, proapoptotic signal-transducing molecules concentrate on mitochondria and promote outer membrane permeabilization (OMP) leading to the release of the cell death effectors [204, 221]. Two plausible mechanisms underpin the mitochondrial OMP [221]. On the one hand, an inner permeability transition pore can be opened by various stimuli, which permits a water flow inside the mitochondrion matrix. Swelling the mitochondrion followed by water influx leads to the outer membrane breakdown. On the other hand, the mitochondrial OMP turns out to be controlled by the direct interaction of proapoptotic BCL-2-family proteins with the outer membrane. Consequently, the mitochondrial OMP causes the release of intermembrane proteins.

For instance, Cyt c release activates apoptosome formation via caspase-dependent processes. The dissociation of Cyt c from high-affinity cardiolipin follows by the Cyt c expulsion [222]. After Cyt c is injected into the cytosol, it rapidly reduces via the cytosolic reductants [204]. Xin et al. [204] have captured the real-time dynamics of Cyt c reduction during apoptosis via intracellular imaging of QBET signal between Cyt c and GNPs (Figure 7d). Since Cyt c mediates the electron transfer between mitochondrial complexes III and IV, its expulsion during apoptosis disrupts the inner-membrane ETC. Complex I will donate electrons to molecular oxygen driving the ROS formation, such as superoxide [219]. Superoxide production tends to promote death of cells susceptible to oxidant stress. Furthermore, the Cyt c absence in the mitochondrial ETC implicates mitochondrial depolarization, provoking exhaustion of ATP production and eventual cell necrosis. Specific proapoptotic proteins, *e.g.*, P66^Shc^, can generate intracellular ROS as apoptosis signaling molecules [212, 220, 221]. P66^Shc^ can oxidize Cyt c by extracting electrons from the mitochondrial ETC followed by the generation of hydrogen peroxide [212, 223]. Ultimately, accumulated hydrogen peroxide initiates cellular apoptosis via PTP opening and mitochondrial matrix swelling.

### 6.4 DNA repair with photolyase

DNA molecules carry information throughout the cell generations and, unlike proteins or RNA molecules, cannot be replaced [224]. Accumulation of mutations of a nucleotide sequence of DNA habitually promotes cancer in mammals. Therefore, DNA repair systems mitigate the cumulative effect of rare DNA damaging reactions, which makes cell life possible. DNA repair systems comprise numerous versions that are conventionally classified as mismatch repair, base-excision repair, nucleotide-excision, and direct repair [224]. A well-characterized example of the DNA direct repair system that involves a specific enzyme “photolyase” relies on electron transfer to restore UV-damaged DNA [225]. Photolyases are flavoenzymes that repair UV-induced DNA damage. Cyclobutane pyrimidine dimers (CPDs) and pyrimidine–pyrimidone (6–4) photoproducts (6–4 PP) are formed as a result of DNA lesions by UV irradiation [226]. CPDs structure is formed via covalent bonding between two thymine bases, while 6–4 PP construction is accompanied by covalent linking between two adjacent pyrimidine bases [227]. CPD photolyase anchors two cofactors: reduced flavin adenine dinucleotide (FADH^−^) and a light-harvesting photoantenna, either 8-hydroxy-5-deazaflavin or 5,10 methenyltetrahydrofolate [228, 229]. CPD photolyase splits the pyrimidine dimer bond by taking advantage of electron transfer processes between photoexcited (FADH^−*^) and CPD [228, 230, 231]. FADH^−^ is excited either directly by light in the blue/near UV range or via the transfer of energy harvested by photoantenna [228, 232, 233]. Photoexcited FADH^−*^ transfers an electron destined to break the dimer-bond. The electron transfer occurs either by direct quantum tunneling or as a two-step hopping via the adenine residue of the photolyase [225]. Consequently, the electron is transferred back to neutral FADH^°^ restoring the initial state of FADH^−^ and catalytic activity of the CPD photolyase [234, 235]. Similarly to the CPD repair process, 6–4 PP photolyase executes the electron transfer processes between FADH^−*^ cofactor and 6–4 PP to repair the corresponding DNA lesion [225]. The repair process is additionally accompanied by a proton transfer between a histidine residue (H364) of the enzyme and 6–4 PP, as well as a series of covalent bond rearrangements leading to the reconstruction of two thymine bases. Experimentally, these enzymatic photorepair processes can be monitored using a femtosecond-resolved spectroscopy [236–238].

## 7 Conclusions

Since the experimental discovery of the strong light–matter coupling phenomenon four decades ago, there have been numerous advances toward increasing coupling strength and observing this phenomenon with various types of optical cavities and two-level systems at room temperature. As we describe here, intriguing phenomena such as hybrid energy state formation and coherent energy transfer between electromagnetic and matter modes stimulate the application of strong coupling in adjacent scientific fields such as biology, chemistry, or quantum information.

Vibro-polaritonic strong coupling enables modification of the chemical reaction landscape and has been developed over the last decade to influence corresponding reactions and create molecular devices. Plexcitonic nanocavities and their unconventional fabrication and manufacturing techniques [239–241] boost strong excitonic effects, leading to controllable catalysis or exciton manipulation in 2D materials. Alteration of the energy transfer in the photosynthesis process, generation of a “living polariton,” and control over the electron transfer in metabolic pathways open exciting avenues for research in quantum life sciences. The use of strong coupling in quantum biology is in its infancy, and its potential for interrogating biological processes has attracted interest for future exploration in life sciences and transformative medicine.
